# Phase I/II study of gefitinib (Iressa^®^) and vorinostat (IVORI) in previously treated patients with advanced non-small cell lung cancer

**DOI:** 10.1007/s00280-014-2664-9

**Published:** 2015-01-01

**Authors:** Ji-Youn Han, Soo Hyun Lee, Geon Kook Lee, Tak Yun, Young Joo Lee, Kum Hui Hwang, Jin Young Kim, Heung Tae Kim

**Affiliations:** Center for Lung Cancer, Research Institute and Hospital, National Cancer Center, 323 Ilsan-ro, Ilsandong-gu, Goyang-si, Gyeonggi-do 410-769 Republic of Korea

**Keywords:** Gefitinib, Vorinostat, EGFR, NSCLC

## Abstract

**Purpose:**

Vorinostat has been shown to overcome resistance to gefitinib. We performed a phase I/II study combining gefitinib with vorinostat in previously treated non-small cell lung cancer (NSCLC).

**Methods:**

A 3 + 3 dose-escalation design was used to determine maximum tolerated dose (MTD) and recommended phase II dose (RP2D). Three dose levels were tested: 250 mg/day gefitinib on days 1–28 and 200, 300 or 400 mg/day vorinostat on days 1–7, and 15–21 out of every 28 days. The primary endpoint was median progression-free survival (PFS).

**Results:**

Fifty-two patients were enrolled and treated (43 in phase II). The median age was 59 years, 28 patients were male, 44 had adenocarcinoma, 29 had never smoked, and 36 had undergone one prior treatment. Twenty-two patients exhibited sensitive EGFR mutations. Planned dose escalation was completed without reaching the MTD. The RP2D was 250 mg gefitinib and 400 mg vorinostat. In 43 assessable patients in phase II, the median PFS was 3.2 months; the overall survival (OS) was 19.0 months. There were 16 partial responses and six cases of stable disease. In EGFR-mutant NSCLC, response rate was 77 %, median PFS was 9.1 months, and median OS was 24.1 months. The most common adverse events were anorexia and diarrhea.

**Conclusions:**

Treatment with 250 mg gefitinib daily with biweekly 400 mg/day vorinostat was feasible and well tolerated. In an unselected patient population, this combination dose did not improve PFS. However, this combination showed a potential for improving efficacy of gefitinib in EGFR-mutant NSCLC (NCT01027676).

## Introduction

Epidermal growth factor receptor (EGFR)–tyrosine kinase inhibitors (TKIs) are now the therapy of choice for patients with EGFR-mutant non-small cell lung cancer (NSCLC). However, despite the initial response to EGFR-TKIs, most patients develop resistance and eventually relapse. The mechanisms responsible for acquired resistance to EGFR-TKIs include secondary EGFR T790 M mutation, MET amplification, epithelial-to-mesenchymal transition (EMT) signature, histologic transformation to small cell lung cancer, and AXL kinase activation [1]. Furthermore, approximately 20 % of patients harboring sensitive EGFR mutations exhibit a suboptimal response or primary resistance to EGFR-TKIs. In addition to the coexistence of other genetic alterations, such as PIK3CA or EGFR exon 20 insertion mutations, germline BIM deletion polymorphism has been reported as a possible mechanism of primary resistance to EGFR-TKIs [2]. Novel strategies to overcome the multifactorial resistance are needed to improve the efficacy of EGFR-TKIs, particularly in patients with EGFR mutations.

In addition, there is controversy regarding the use of EGFR-TKIs for patients with EGFR wild-type NSCLC. So far, EGFR-TKIs have been widely used for advanced NSCLC irrespective of the EGFR mutation status because earlier trials that demonstrated the efficacy of EGFR-TKIs for second- or third-line therapy of advanced NSCLC did not consider EGFR genotype. However, recent randomized trials comparing erlotinib and docetaxel as a second-line therapy in EGFR wild-type NSCLC demonstrated the clear superiority of docetaxel over EGFR-TKIs in patients with EGFR wild-type NSCLC [3, 4]. Thus, further defining the subpopulation of EGFR wild-type NSCLC patients that is suitable for EGFR-TKIs therapy is needed.

Histone deacetylase inhibitors (HDACis) have emerged as promising multifunctional anticancer agents that regulate gene expression and transcription through chromatin remodeling. HDACis can also modulate a variety of cellular functions, including growth, differentiation, and survival, through the acetylation of a wide range of proteins, including transcription factors, molecular chaperones, and structural components [5]. Recent data suggest that HDACis can increase sensitivity to EGFR-TKIs in lung cancer cells. HDACis can reverse resistance to EGFR-TKIs through induction of E-cadherin expression in lung cancer cells [6]. In addition, HDACis induce acetylation of Hsp90, resulting in reduced association of Hsp90 with key chaperone proteins, including EGFR, c-Src, STAT3, and Akt [7]. Furthermore, HDACis increase the expression of the proapoptotic BH3 domain-containing isoform of BIM, which restores the sensitivity to EGFR-TKIs [8]. Thus, current research on incorporating HDACis in NSCLC treatment is focused on the combination of HDACis with EGFR-TKIs [9].

Vorinostat is an inhibitor of class I and II histone deacetylases that regulate the transcription of various genes involved in cell survival and apoptosis. Vorinostat has demonstrated profound anti-growth activity against NSCLC cells [10]. Given the potential synergy between HDACis and EGFR-TKIs, we conducted a phase I/II study of gefitinib and vorinostat in patients with advanced NSCLC.

## Methods

### Patients

The main eligibility criteria were histologic confirmation of advanced NSCLC, previous chemotherapy with at least one platinum-containing regimen, age ≥ 18 years, an Eastern Cooperative Oncology Group (ECOG) performance status (PS) of less or equal to 2, and a measurable disease according to the Response Evaluation Criteria in Solid Tumors (RECIST). Adequate hematologic (WBC count ≥4,000/mm^3^, platelet count ≥150,000/mm^3^), hepatic (bilirubin level ≤1.5 mg/dL, AST/ALT ≤80 IU/L), and renal (creatinine concentration ≤1.5 mg/dL) function was required. Patients with brain metastases were enrolled if they were clinically stable without steroid treatment. The exclusion criteria included serious concomitant systemic diseases or a history of uncontrolled cardiac dysfunction, or any previous treatment with EGFR signaling inhibitors or HDAC inhibitors. The protocol was approved by an independent ethics committee/institutional review board and was conducted in accordance with the Declaration of Helsinki, Good Clinical Practice. Each patient provided written informed consent.

### Study design

The phase I study was a standard 3 + 3 dose-escalation design, followed by a phase II part. The primary endpoint of the phase I part was to determine the maximum tolerated dose (MTD) and the recommended phase II dose (RP2D) of vorinostat in combination with gefitinib. The primary endpoint of phase II part was progression-frees survival. In the phase I study, three patients were treated per cohort for one cycle (28 days per cycle). Dose-limiting toxicity (DLT) was defined as any grade 3 or 4 non-hematologic toxicity (except nausea or vomiting that responds to symptomatic therapy, fatigue that responds to maximal management and alopecia) or any grade 4 hematologic toxicity occurring during the first cycle. Toxicity was graded according to the National Cancer Institute Common Toxicity Criteria of Adverse Events (CTCAE) version 4.0. In the absence of any DLT, three patients were treated in the subsequent cohort. The presence of DLT in a cohort required that another three patients be treated in the cohort for one cycle. If no DLTs occurred, then dose escalation continued. RP2D was defined as the highest dosage at which one of six patients at most experienced a DLT. No intra-patient dose escalation was permitted.

### Treatment delivery

All patients received once-daily oral doses of 250 mg gefitinib on days 1–28 in combination with vorinostat on days 1–7 and days 15–21 of each 28 days cycle. Up to three dose levels of vorinostat were evaluated (200, 300, and 400 mg/day). During phase II, vorinostat was administered at the RP2D of 400 mg/day. Study treatment continued until disease progression (PD) or until another termination criterion was met: unacceptable toxicity, consent withdrawal, loss to follow-up, death, major protocol violation, or noncompliance.

### Study assessment

Safety assessment included history, physical examinations, vital signs, ECOG PS, adverse events (AEs), electrocardiography (ECG), blood chemistry, and hematology. Safety assessments were performed at screening, biweekly (days 1 and 15) during cycles one and two, on day 1 of subsequent cycles, and during the final study visit.

Baseline computed tomography (CT) scans of the chest and abdomen, bone scintigraphy, and brain magnetic resonance imaging or CT were obtained within 4 weeks before initiation of treatment. Efficacy variables, including progression-free survival (PFS) and overall survival (OS), were evaluated during phase II. Tumor response was assessed using RECIST 1.1 [11] after every two cycles of therapy.

### EGFR and KRAS mutation analysis

Genomic DNA was extracted from 10 % neutral formalin-fixed, paraffin-embedded (FFPE) tumor tissue blocks using the QIAamp DNA Mini Kit (QIAGEN, Hilden, Germany). We analyzed *EGFR* and KRAS mutations using the polymerase chain reaction (PCR)-based direct DNA sequencing method [12].

### Genotyping of BIM deletion polymorphism (BIM DEL)

We obtained blood samples before treatment. We extracted genomic DNA from patients’ peripheral blood and genotyped the deletion by a single PCR reaction using the primers 5′-ccaccaatggaaaaggttca-3′ and 5′-gcctgaaggtgctgagaaag-3′ and Hotstartaq DNA Polymerase (Qiagen) with the following thermo-cycling conditions: 94 °C for 15 min, (94 °C for 15 s, 60 °C for 30 s, and 68 °C for 5 min) × 35, and 68 °C for 10 min. The resulting PCR products from the deletion (970 bp) and the wild-type (3,873 bp) alleles were analyzed on 1.5 % agarose gels. We performed two separate PCR reactions to determine the presence of the wild-type and deletion alleles.

### Immunohistochemistry for E-cadherin and vimentin

Samples with E-cadherin (Zymed, CA, USA) and vimentin (Ventana, AZ, USA) immunohistochemistry staining intensity scores of 0 or +1 (<50 % of the cells have complete circumferential membrane staining at a low intensity) were classified as negative. Those with +2 or +3 (≥50 % of the cells have complete circumferential membrane staining at a high intensity) were classified as positive.

### Statistical analysis

The primary object of the phase I study was to determine the MTD of vorinostat in combination with the standard dose of gefitinib. The last six patients enrolled into the phase I study were included in the analysis of the phase II study. The primary endpoint of the phase II component was to reject the null hypothesis (median PFS, 3.5 months) and to accept the alternative hypothesis (median PFS, 6.5 months). To test the hypothesis and to calculate the sample size, we assumed an exponential distribution. Thus, the estimated 6-month PFS under the null hypothesis and alternative hypothesis were 0.305 and 0.530, respectively. To achieve a power of .80, with *α* = .05, we calculated a sample size of 40 patients. Anticipating a 10 % dropout rate, the samples size for the phase II portion totaled 44 patients.

All tests of hypotheses were conducted at a two-sided *α* = 0.05 level. The log-rank test was used to compare PFS and OS time according to mutation status. The distribution of PFS and OS were estimated using the Kaplan–Meier method. Statistical comparison of the response rates according to the mutation status was performed using Chi-squared or Fisher’s exact test.

## Results

### Patient characteristics

Between July 2010 and June 2013, 52 patients were enrolled in this study. The patient characteristics are summarized in Table 1. All patients had stage IV disease, and most of the patients exhibited good PS and adenocarcinoma histology. Thirty-six patients received the study treatment as second-line therapy. The most common reason for study discontinuation was disease progression (45 of 52: 86.5 %).

**Table 1:** Patient characteristics

	Phase I			Phase II
	Level 1	Level 2	Level 3	
*N*	3	6	6	37
Median age (range)	67 (65–76)	63.5 (59–71)	52.5 (44–66)	56 (39–79)
Male/female	2/1	3/3	2/4	21/16
Stage IIIB/IV	0/3	0/6	0/6	0/37
ECOG performance status 0/1/2	0/3/0	1/2/3	2/4/0	5/25/7
Histology, adenocarcinoma/squamous/other^a^	3/0/0	4/2/0	5/1/0	32/3/2
Sensitive EGFR mutation, positive/negative/unknown	1/2/0	4/2/0	4/2/0	13/23/0
K-RAS mutation, positive/negative/unknown	1/0/2	1/4/1	0/6/0	3^b^/34/0
BIM deletion polymorphism, positive/negative	1/2	1/5	1/5	4/33
Prior chemotherapy regimen, one/two	2/1	3/3	5/1	26/11

^a^NOS, sarcomatoid carcinoma

^b^One patient had concurrent EGFR exon 19 deletion with A871T and KRAS G12S mutations: This patient is regarded as KRAS mutation positive

### Phase I study results

The toxicities encountered during the phase I study are summarized in Table 2. Three patients were enrolled in the level 1 treatment, and none experienced DLT. Three patients were enrolled in the level 2 treatment, and one experienced DLT due to grade 3 hyperglycemia. Consequently, another three patients were enrolled in the level 2 treatment, and none experienced DLT. In the level 3 treatment group, the initial three patients experienced no DLT. Thus, the level 3 group was expanded up to six patients, and one experienced DLT due to grade 3 anorexia. The RP2D was determined to be biweekly 400 mg/day vorinostat in combination with daily 250 mg gefitinib (level 3).

**Table 2:** Toxicities in phase 1 (*n* = 15)

	Level 1 (*n* = 3)				Level 2 (*n* = 6)				Level 3 (*n* = 6)			
Grade	1	2	3	4	1	2	3	4	1	2	3	4
Anemia	2	1	0	0	2	1	0	0	1	1	0	0
Blood bilirubin increased	0	0	0	0	1	0	0	0	1	0	0	0
Creatinine increased	0	0	0	0	2	0	0	0	0	0	0	0
Hyperglycemia	1	1	0	0	2	0	1	0	1	0	0	0
Neutrophil count decreased	0	0	0	0	0	0	0	0	0	1	0	0
Platelet count decreased	0	0	0	0	1	0	0	0	1	0	0	0
White blood cell count decreased	0	0	0	0	0	0	0	0	3	0	0	0
Anorexia	1	0	0	0	2	0	0	0	2	1	1	0
Diarrhea	0	0	0	0	4	0	0	0	5	1	0	0
Dry mouth	0	0	0	0	1	0	0	0	1	0	0	0
Dry skin	0	0	0	0	2	0	0	0	2	0	0	0
Fatigue	1	0	0	0	3	0	0	0	1	0	0	0
Oral mucositis	0	0	0	0	2	0	0	0	4	1	0	0
Nausea	1	0	0	0	1	0	0	0	3	0	1	0
Papulopustular rash	0	0	0	0	0	0	0	0	1	0	0	0
Pruritus	0	0	0	0	3	0	0	0	4	0	0	0
Acneiform rash	0	0	0	0	1	0	0	0	1	0	0	0
Maculopapular rash	0	0	0	0	3	0	0	0	3	0	0	0
Nasal mucositis	0	0	0	0	0	0	0	0	1	0	0	0
Vomiting	0	0	0	0	1	0	0	0	2	0	0	0

### Phase II study

The following analysis is based on 43 patients, including six patients who were treated during the MTD of the phase I portion and 37 patients who were treated during the phase II portion of this study. The follow-up data were frozen on March 3, 2014. The median follow-up was 16.2 months (95 % CI 13.2 to 19.3 months). The median number of cycles was two (range 1–24 cycles).

### Response and survival

**Fig. 1:**
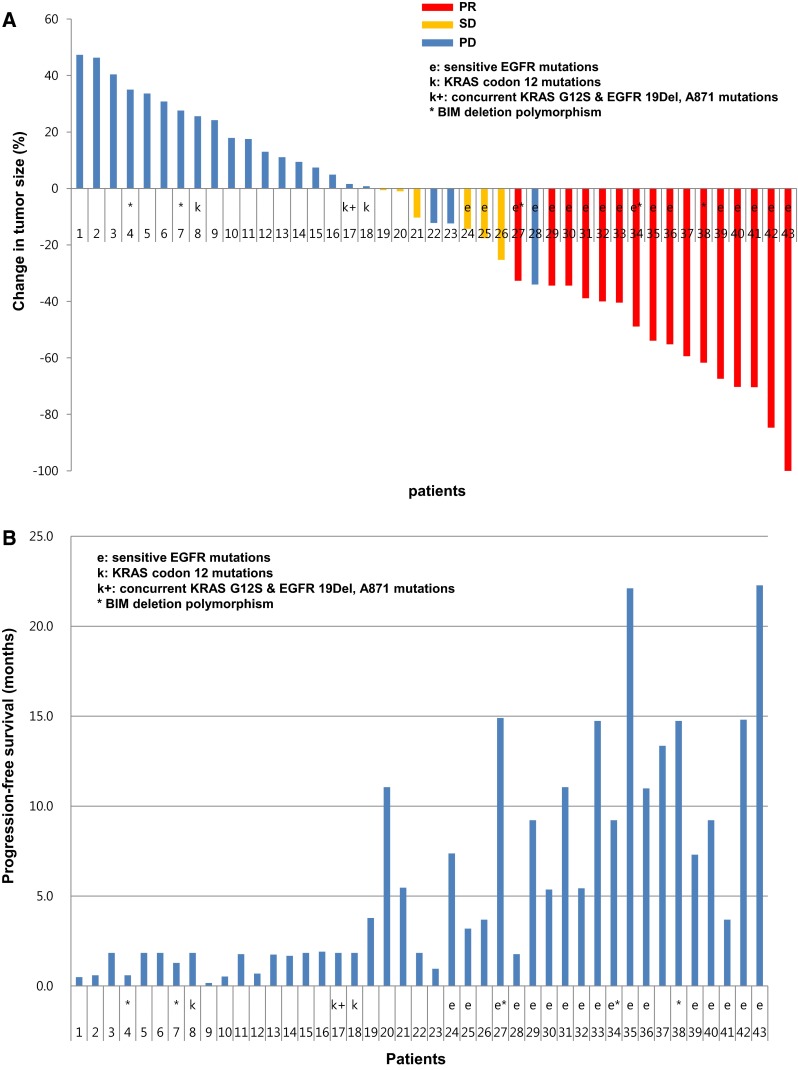
Efficacy. **a** Waterfall plot of response. **b** Progression-free survival PR partial response; SD stable disease; PD progressive disease; e sensitive EGFR mutations; k KRAS codon 12 mutations; BIM Del BIM deletion polymorphism

Among 43 patients, there were 16 patients with a partial response (37.2 %), 6 with stable disease (14.0 %), and 21 with progressive diseases (48.8 %). The median PFS was 3.2 months (95 % CI 2.3 to 4.1 months). The median OS was 19.0 months (95 % CI 17.2 to 20.8 months). The response and PFS data are presented in Fig. 1a, b.

### Toxicity

Toxicity was assessable in all 43 patients (Table 3). The most frequent grade 3 toxicities were anorexia (11.6 %), diarrhea (9.3 %), fatigue (7.0 %), and anemia (4.7 %). There was no grade 4 hematologic toxicity. There was no treatment-related death or irreversible toxicity that was considered to be related to the treatment in this study.

**Table 3:** Toxicities in phase II (*n* = 43)

	Grade				%
	1	2	3	4	Grade 3/4 (total)
Diarrhea	19	12	4	0	9.3 (81.4)
Anorexia	12	14	5	0	11.6 (72.1)
Pruritus	21	5	0	0	0 (60.5)
Acneiform rash	16	10	0	0	0 (60.5)
Hyperglycemia	19	4	0	0	0 (53.5)
Anemia	12	8	2	0	4.7 (51.2)
Vomiting	13	6	1	0	2.3 (46.5)
Nausea	13	4	1	0	2.3 (41.9)
Fatigue	12	2	3	0	7.0 (39.5)
Oral mucositis	12	2	0	0	0 (32.6)
White blood cell count decreased	12	0	0	0	0 (27.9)
Dry skin	10	1	0	0	0 (25.6)
Hypocalcemia	8	2	0	0	0 (23.3)
ALP increased	9	1	0	0	0 (23.3)
Platelet count decreased	9	0	1	0	2.3 (23.3)
Creatinine increased	10	0	0	0	0 (23.3)
ALT increased	6	2	1	0	2.3 (20.9)
AST increased	6	2	0	0	0 (18.6)
Weight loss	4	4	0	0	0 (18.6)
Headache	4	4	0	0	0 (18.6)
Blood bilirubin increased	5	2	0	0	0 (16.3)
Hyponatremia	6	0	1	0	2.3 (16.3)
Electrocardiogram QT corrected interval prolonged	6	0	0	0	0 (14.0)
Maculopapular rash	6	0	0	0	0 5 (14.0)
Epigastric pain	0	6	0	0	0 (14.0)
Abdominal pain	2	1	3	0	7.0 (14.0)
Hypophosphatemia	0	5	1	0	2.3 (14.0)
Hypokalemia	5	0	1	0	2.3 (14.0)
Constipation	4	1	0	0	0 (11.6)
Peripheral sensory neuropathy	4	1	0	0	0 (11.6)
LDH increased	5	0	0	0	0 (11.6)
Hypermagnesemia	0	0	3	0	7.0 (7.0)
Neutrophil count decreased	0	4	0	0	0 (9.3)
Dizziness	2	1	1	0	2.3 (9.3)
Nasal mucositis	3	1	0	0	0 (9.3)
Paronychia	1	0	1	0	2.3 (4.7)
Dehydration	0	0	1	0	2.3 (2.3)
Hand foot syndrome	0	1	0	0	0 (2.3)
Acute renal failure	0	0	1	0	2.3 (2.3)

### Exploratory biomarker analysis

**Fig. 2:**
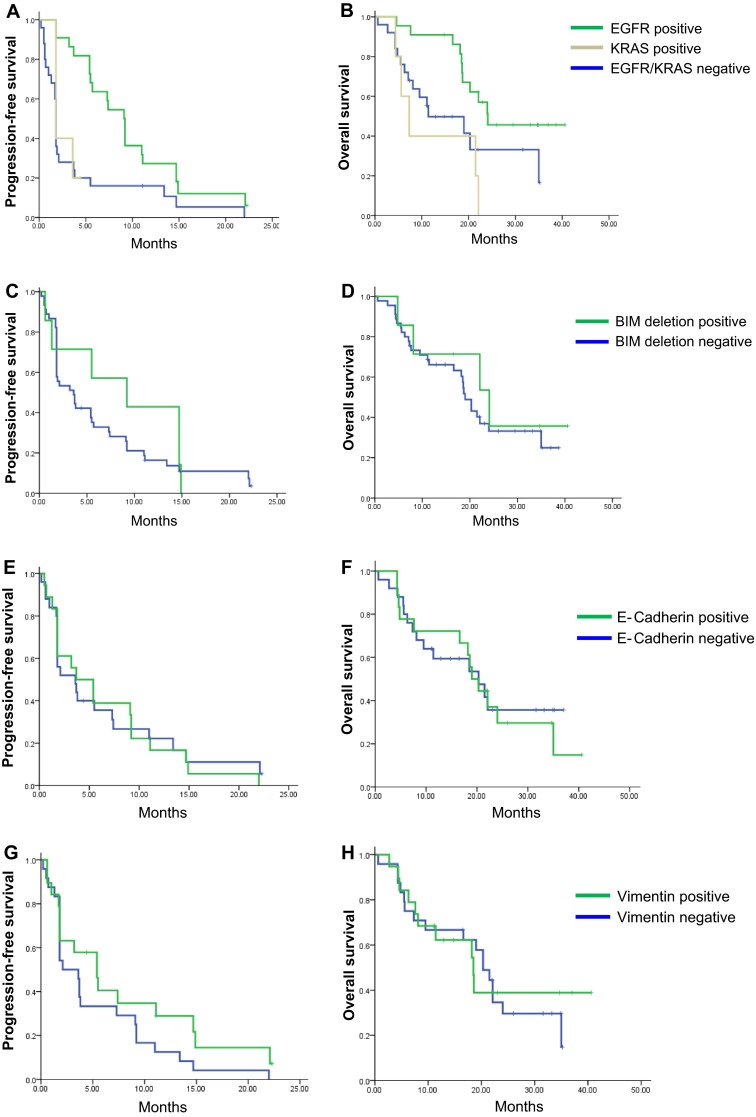
Progression-free and overall survival according to EGFR and KRAS mutation status (**a**, **b**), BIM deletion polymorphism (**c**, **d**), E-cadherin expression (**e**, **f**), and vimentin expression (**g**, **h**), respectively

We analyzed EGFR and KRAS mutations, as well as BIM DEL, and E-cadherin and vimentin expression in all patients. Of the 52 patients enrolled in this study, 22 exhibited sensitive EGFR mutations (sixteen exon 19 deletions, five L858R mutations, and one R776H mutation) and five exhibited KRAS mutations (three G12C and two G12S mutations). One patient with KRAS G12S exhibited a concurrent EGFR exon 19 deletion and A871T mutation. This case was classified as KRAS mutation positive. BIM DEL was observed in seven patients. Immunohistochemistry was available in 43 patients, and eighteen and nineteen patients exhibited positive E-cadherin and vimentin expression, respectively. The response and survival data according to biomarkers are summarized in Table 4. The presence of sensitive EGFR mutations was predictive of higher response rates (RR) and longer PFS and OS compared with patients with KRAS mutations or patients without either mutation (Fig. 2a, b). Patients harboring BIM DEL mutations exhibited a trend toward higher RR and longer PFS; however, the trend failed to achieve statistical significance (Fig. 2c, d). E-cadherin and vimentin expression was not associated with RR or survival (Fig. 2e–h).

**Table 4:** Exploratory biomarker analysis

	EGFR/KRAS mutation			BIM deletion polymorphism		E-cadherin		Vimentin	
	EGFR pos	KRAS pos	Both neg	Pos	Neg	Pos	Neg	Pos	Neg
*N*	22	5	25	7	45	18	25	19	24
Response									
PR	17	0	3	5	15	9	7	7	9
SD	3	1	4	0	8	2	5	4	3
PD	2	3	18	2	21	7	12	7	12
NA	0	1	0	0	1	0	1	1	0
P*	<0.0001			0.247		0.426		0.541	
PFS, months									
Median	9.1	1.8	1.8	9.2	3.6	3.6	3.6	5.4	2.1
95 % CI	7.4–10.8	NA	1.7–1.9	0.0–18.7	1.2–6.0	0.7–6.7	0.5–6.7	2.4–8.4	1.1–3.1
*P* ≠	0.004			0.486		0.842		0.125	
OS, months									
Median	24.1	7.3	11.4	24.1	19.0	19.0	20.3	18.5	20.3
95 % CI	NA	3.7–11.0	0.0–23.1	6.7–41.5	17.0–21.0	15.5–22.5	8.1–32.5	10.8–26.2	16.5–24.1
*P* ≠	0.017			0.561		0.843		0.812	

*pos* positive, *neg* negative, *NA* not assessable, *PFS* progression-free survival, *OS* overall survival, *CI* confidence interval

* Chi-square or Fisher’s exact test, ≠ Kaplan–Meier test

## Discussion

Our study demonstrated that gefitinib administered daily at a dose of 250 mg with biweekly vorinostat at a dose of 400 mg/day was feasible and well tolerated. However, the efficacy results measured by PFS do not support this combination for molecularly unselected NSCLC patients. Nevertheless, it is noteworthy that patients harboring sensitive EGFR mutations achieved a remarkable RR of 77 % and a median PFS of 9.1 months even in the second- or third-line setting, results that are comparable to the first-line use of gefitinib in EGFR-mutant NSCLC.

Thus far, four randomized phase III studies have demonstrated superior RR and PFS with first-line use of gefitinib over platinum-based chemotherapy in EGFR-mutant NSCLC. First-line gefitinib in EGFR-mutant NSCLC yields consistent RR of 70–80 % and a median PFS of 9–10 months [13–16]. To date, there are no direct comparisons between first-line versus second-line use of EGFR-TKIs in EGFR-mutant NSCLC. Although the sequence of EGFR-TKIs in EGFR-mutant NSCLC may not affect the OS [17], the tumor response rates to second-line EGFR-TKIs are usually lower than to first-line use. Maemondo et al. [15] documented that the RR to gefitinib was slightly worse in the second-line setting compared with the first-line setting (58.5 vs. 73.7 %) in a randomized phase III study. Sugio et al. [18] also reported that the RR to gefitinib was lower in the second-line setting compared with the first-line setting (50.0 vs. 77.8 %). In a randomized phase III study that compared gefitinib with docetaxel as a second- or third-line therapy, the gefitinib arm demonstrated a RR of 42.1 % and a median PFS of 7.0 months in EGFR-mutant patients [19]. Although there was no significant difference in the OS treatment effect, the OS in EGFR-mutant NSCLC was 14.2 months with gefitinib [19]. Some data suggest that the relatively inferior response to second-line EGFR-TKIs may result from the decreased abundance of EGFR-mutant tumor cells after chemotherapy [20, 21].

It is clear that EGFR-TKIs are most active in EGFR-mutant NSCLC. The sensitive EGFR mutations target the TK domain that is essential for the phosphorylation function, which results in enhanced kinase activity and also in increased sensitivity to EGFR-TKIs [22]. A recent experimental study revealed that vorinostat treatment increased the acetylation of EGFR, which leads to enhanced EGFR phosphorylation in cancer cells. Additionally, combination therapy effectively inhibited cell growth in vitro compared with individual therapy [23]. Despite second- or third-line use of gefitinib in our study, the relatively higher RR and longer survival times observed in EGFR-mutant NSCLC may be attributed to the combination with vorinostat. Vorinostat-induced acetylation-enhanced tyrosine kinase phosphorylation of EGFR may overcome or delay the development of acquired resistance to gefitinib caused by the low abundance of EGFR-mutant cancer cells. By contrast, the efficacy observed in patients with wild-type EGFR was similar to other reports. Accordingly, vorinostat in combination with gefitinib may be more effective for patients with EGFR-mutant NSCLC. At the time of the study initiation, EGFR mutation test was not routinely performed in Korea and gefitinib was usually used as second- or third-line therapy. Thus, we enrolled non-molecularly selected patients.

Very recently, Reguart et al. [24]. reported a phase I/II study of vorinostat and erlotinib for EGFR-mutant NSCLC after erlotinib progression. The authors found no meaningful activity in the erlotinib-resistant population. Given that the most common cause of acquired resistance to EGFR-TKIs in EGFR-mutant NSCLC is T790 M mutation, vorinostat may be not sufficient to overcome T790 M-related resistance to EGFR-TKIs. Witta et al. [25] reported a randomized phase II trial of erlotinib with or without entinostat, an isoform-selective HDACis, in previously treated NSCLC. The authors also failed to demonstrate improved outcomes. Nevertheless, the authors reported that high E-cadherin expression was predictive of longer OS with the combination therapy. However, biomarker analysis was available in only a subset of patients, and more patients enrolled in the combination arm were EGFR FISH positive. Thus, the impact of E-cadherin expression on HDACis in combination with erlotinib remains unclear. We also analyzed E-cadherin expression. Furthermore, we added vimentin expression to further define cases with an epithelial phenotype. However, we did not observe any significant association with efficacy. We also analyzed the predictive role of BIM DEL. BIM DEL has been reported to be associated with intrinsic resistance to EGFR-TKIs due to the impaired generation of BIM with the proapoptotic BH3 domain [2]. However, vorinostat can upregulate the expression of BIM protein with BH3 domain, which restore sensitivity to gefitinib in EGFR-mutant NSCLC cells with BIM DEL [8]. Patients with BIM DEL exhibited a trend toward longer PFS compared with those without BIM DEL. However, only seven (13 %) of 52 patients exhibited BIM DEL; thus, the impact of vorinostat in combination with gefitinib in this population should be investigated further.

The combination therapy of vorinostat and gefitinib as second- or third-line therapy did not improve outcomes in an unselected patient population. Nevertheless, the planned biomarker analysis suggests that this combination may be effective for EGFR-mutant NSCLC by enhancing EGFR phosphorylation. Although further study is required to confirm the usefulness of vorinostat in combination with gefitinib in EGFR-mutant NSCLC, our study suggests the potential benefit of vorinostat for improving the efficacy of EGFR-TKIs in EGFR-mutant NSCLC.
